# Accuracy of estimating total-tract fibre and protein fractions digestibilities using uNDF, uNDFom, and AIA markers, and their NIRS prediction potential in dairy sheep and goats

**DOI:** 10.1371/journal.pone.0331549

**Published:** 2025-09-22

**Authors:** Tommaso Danese, Alberto Guerra, Marica Simoni, Giorgia Mantovani, Arianna Goi, Rosario Pitino, Alexandros Mavrommatis, Massimo De Marchi, Federico Righi, Eleni Tsiplakou

**Affiliations:** 1 Department of Veterinary Science, University of Parma, Parma, Italy; 2 DAFNAE, University of Padova, Legnaro, Italy; 3 Laboratory of Nutritional Physiology and Feeding, Department of Animal Science, Agricultura University of Athens, Athens, Greece; Tokat Gaziosmanpaşa University: Tokat Gaziosmanpasa Universitesi, TÜRKIYE

## Abstract

This study investigated the use of uNDF, uNDFom, and acid insoluble ash (AIA) markers for estimating the total-tract and total-tract apparent digestibility (ttD and ttaD) of nutrients in dairy sheep and goats fed alfalfa hay and concentrate separately, and the potential of Near-Infrared Spectroscopy (NIRS) in predicting the estimated digestibility. A total of 180 faecal samples were collected from animals fed alfalfa hay and concentrate at varying ratios (F:C – 60:40, 50:50, 40:60). The samples underwent wet chemistry markers and nutrients analysis, to evaluate the digestibility of dry matter (DM), crude protein (CP), soluble CP, ash, neutral detergent fibre (NDF), acid detergent fibre (ADF), hemicellulose, cellulose, neutral detergent insoluble CP (NDICP), acid detergent insoluble CP (ADICP), and potentially degradable NDF (pdNDF). NIRS spectra acquisition was performed on the same samples and predictive models developed and tested. Compared to digestibility determined by total collection, results indicate that all the considered markers, namely uNDF, uNDFom and AIA tend to underestimate ttaD and ttD. Among the three makers, AIA resulted in the lowest recovery. Using uNDF as marker, NIRS predictive models showed almost adequate screening performance for ttADICPD and ttAshD, with a R²_ExV_ values of 0.63 and 0.59, and RPD_ExV_ of 1.56 and 1.45, respectively. The uNDFom marker showed better performance for ttaDMD and ttADICPD preliminary screening, with R^2^_ExV_ values of 0.55 and 0.62, and RPD_ExV_ values of 1.42 and 1.61, respectively. The study highlights that all the considered markers underestimate the nutrients digestibility, while uNDFom performed better concerning the NIRS calibration. Despite the encouraging results obtained, the NIRS accuracy in predicting digestibility traits in small ruminants remains poor, and further research are needed to explore its potential for nutrients ttD and ttaD measurement in sheep and goats.

## Introduction

Animal feeds and diets are characterized by different amounts of rapidly, slowly available, or unavailable nutrients. Rapidly available nutrients provide fast energy, while slowly available nutrients are limiting both the amount of energy released to the animals and, along with the unavailable pools, the dry matter intake (DMI). Thus, the determination of the amount and, when appropriate, digestibility, of NDF, ADF, uNDF and ADL -needed for the calculation of their components hemicellulose and cellulose and the potentially digestible NDF (pdNDF)- as well as of their bound nitrogen (neutral detergent insoluble nitrogen – NDIN – and acid detergent insoluble nitrogen – ADIN), are key factors in determining the nutritional value of the diets and livestock’s environmental impact. Indeed, fibre-bound protein is partially lost in manure and environment [1]. Moreover, because of their characteristics, the unavailable pools can be employed as markers for the estimation of the in vivo digestibility of the diets since they include the undigestible NDF (uNDF), which is a promising internal marker for in vivo digestibility estimation [2,3], namely the total-tract apparent digestibility (ttaD) and total tract digestibility (ttD) of various nutrients [4,5]. This marker is particularly useful under field conditions when feed intake and total faecal excretion data are not available [6] and can be refined through correction for ash as uNDFom.

However, the in vitro uNDF and uNDFom determination, as occurs with other feed components, is expensive and time demanding since it involves a 240-h ruminal fermentation followed by aNDF extraction [7] and possibly, as in the case of uNDFom, the determination of the ash on the residue. Similarly, other internal markers, like acid insoluble ash (AIA), require chemical analysis. Therefore, a reliable and rapid technique, such as the NIRS, for the determination of digestibility values could be of interest for the precision feeding implementation.

Under intensive livestock conditions, where the diet formulas are known, NIRS technology can be applied to investigate the digestion process either through the combined analysis of diets and faeces or directly on diet or faecal samples [8–10]. The NIRS has been tested for the estimation of some faecal chemical traits in dairy cows [5] and to predict the digestibility of beef cattle and buffalo diets fed total mixed ration [3,11] with some interesting and critical results. In work on beef cattle, only faecal NDF and ADF (%DM) were satisfactory estimated by NIRS and while studying the NIRS prediction of digestibility, it was concluded that the prediction model developed cannot be recommended for the estimation of any digestibility parameter [3]. In a study on buffalo, the fat, NDIN, and ADIN digestibility were satisfactory estimated by NIRS when using uNDF as an internal marker, and only fat was well determined by NIRS when using AIA for calibration purposes [11].

Only few research studies are available on small ruminants, and their faeces have been studied mainly for the retrospective quantification of feed intake and the investigation of their dietary preferences under grazing conditions in extensive farming systems [12]. It is well known that the calibration of NIRS devices requires reference values that are sometimes difficult to obtain, as in the case of in vivo digestibility. To the best of our knowledge, no research exists on the use of uNDF, uNDFom and AIA as internal markers for digestibility estimation in small ruminants, and specifically in dairy sheep and goats, which are usually fed forages and concentrate separately, under traditional farming systems. Additionally, no research has been done on the possibility to use NIRS to develop predictive model for digestibility measurements estimated by markers in these animals.

Therefore, the aim of this study was: (i) to assess the accuracy of the estimation of fibre and protein fractions ttaD and ttD using uNDF, uNDFom and AIA markers and (ii) to test the effectiveness of NIRS in predicting the same digestibility parameters from faeces of dairy sheep and goats fed forage and concentrate separately.

## Materials and methods

Animal handling, housing, and care followed the Ethical Committee guidelines of the Department of Animal Science of the Agricultural University of Athens (EU 63/2010; Council of the European Union 2010), which owned the animals and gave the written informed consent for their use.

### Sample collection and chemical analysis

A total of 180 fresh, individual faecal samples (300 g) were obtained from Greek crossbreed dairy sheep (n = 8) and goats (n = 4) raised at the Department of Animal Science of the Agricultural University of Athens (Athens, Greece). Sampling and measurements were described in previous work focusing on NIRS calibration based on digestibility determined by total faecal collection [13].

Briefly, the animals were followed during the whole lactation period and fed diets including alfalfa hay (91.8% DM, 51.3% aNDFom, 6.37% ADL, 13.4% CP) and concentrate (91.3% DM, 19.3% aNDFom, 2.33% ADL, 41.2% Starch, 16.8% CP) at ratios of 40:60, 50:50 and 60:40 during the first, second and third phase of the lactation. At the end of each lactation phase, the animals were placed in metabolic cages where, after a 3 days adaptation period, were subjected to measurements. Forages were supplied through basket hay feeders including a net to minimize sorting behaviour. Forage and concentrates were fed separately, once a day and their individual intake was measured by difference between the amounts offered and orts, for 5 days, corresponding to the faecal collection period. Specifically, individual daily total faecal collection was performed to determine the reference digestibility values; the 24 h pool of faeces was thoroughly mixed and subsamples were obtained, dried in an oven at 55°C for 48 h, and stored under vacuum at 4°C for further analysis. The dry matter intake was used to calculate the composition of the diet ingested by individual animals. The diets fed were evaluated by the Small Ruminant Nutrition System (SRNS) based on the structure of the Cornell Net Carbohydrate and Protein System [14,15] and were found to cover the animals’ nutritional requirements.

For the analysis, faecal samples and dietary ingredients were ground in a hammer mill (Retsch S/S Cross Beater Hammer Mill Sk1, Haan, Germany) to pass a 1-mm screen. The DM was determined on an aliquot of 5 g of dried sample which was dried in an oven at 103°C overnight. Fibre fractions were analysed according to [16]. The aNDFom was determined using heat-stable amylase without sodium sulphite and expressed exclusive of residual ash; ADFom was also expressed exclusive of residual ash. A repetition of the fibre fractions analysis was performed to collect the aNDF and ADF residues for fibre bound N determination. The CP content of each sample as well as the NDIN and ADIN were determined by sample combustion digestion at 900°C in excess of oxygen by Dumatherm^®^ (Gerhardt GmbH &Co, Königswinter, Germany) based on the literature [17]. A further repetition of the fibre fractions analysis was performed following the sequential method for hemicellulose and cellulose calculation according to [18].

A 240-h *in vitro* fermentation was performed according to the literature on the topic [19] to measure the proportion of the uNDF in feeds and faeces. Rumen fluid was collected directly from the rumen content of 4 dairy cows at the slaughterhouse and handled as described in a previous work [20]. Briefly, the pooled rumen fluid was blended and filtered under anaerobic conditions at 39.0°C through 4 layers of cheesecloths to obtain the inoculum for the fermentation process. The inoculum was mixed at the ratio 1:4 to the buffer, introduced in flasks containing 0.5 g of sample and incubated in an *in vitro* batch fermentation system [21]. The uNDFom was calculated by subtracting from the uNDF the residual ash after the 240-h fermentation. Ash was determined by ignition at 550°C for 4 h. The CP soluble in NDF was calculated by difference between CP and NDICP. The calculated chemical compositions of the different diets consumed by the animals are shown in Table 1.

**Table 1:** Calculated chemical composition of the diets assumed by the small ruminants offered 3 different forage to concentrate ratios (F:C; 40:60, 50:50 and 60:40 alfalfa hay and concentrate respectively).

	Mean		SD	Min	Max	CV	Mean		SD	Min	Max	CV	Mean			SD	Min	Max
**Diet composition (% DM)**																		
**Alfalfa hay offered (assumed)** ^**a**^	40 (39.9) 60 (60.1)						50 (49.0) 50 (51.0)							60 (58.5) 40 (41.5)				
**Concentrate offered (assumed)** ^**b**^																		
**Diet chemical composition** **(% DM)**																		
**Ash**	5.30	±	0.04	5.18	5.41	0.69	5.45	±	0.05	5.24	5.57	0.95	5.60		±	0.05	5.44	5.64
**CP**	13.1	±	0.06	12.8	13.2	0.49	12.8	±	0.09	12.5	13.1	0.71	12.5		±	0.09	12.4	12.8
**Soluble CP**	10.9	±	0.08	10.6	11.1	0.70	10.6	±	0.11	10.3	11.	1.02	10.2		±	0.10	10.2	10.6
**Ether extract**	1.53	±	0.02	1.46	1.60	1.47	1.44	±	0.03	1.37	1.57	2.19	1.35		±	0.03	1.32	1.44
**aNDFom**	32.4	±	0.72	30.0	34.5	2.22	35.2	±	1.02	31.1	37.6	2.89	38.0		±	0.95	35.0	39.0
**CP-aNDF**	1.21	±	0.02	1.15	1.26	1.65	1.28	±	0.02	1.18	1.34	1.95	1.35		±	0.02	1.28	1.38
**ADFom**	21.2	±	0.65	19.1	23.1	3.04	23.7	±	0.91	20.1	25.8	3.84	26.3		±	0.85	23.5	27.1
**CP-ADF**	0.24	±	0.01	0.20	0.27	4.48	0.28	±	0.01	0.22	0.31	5.40	0.32		±	0.01	0.28	0.34
**Hemicellulose**	11.2	±	0.08	11.1	11.5	0.67	11.5	±	0.11	11.1	11.8	0.92	11.8		±	0.10	11.5	11.9
**Cellulose**	18.8	±	0.54	17.0	20.3	2.88	20.9	±	0.76	17.8	22.7	3.65	23.1		±	0.71	20.8	23.8
**ADL**	2.39	±	0.10	2.05	2.70	4.36	2.80	±	0.15	2.21	3.14	5.27	3.23		±	0.14	2.77	3.35
**Starch**	24.5	±	0.93	21.7	27.5	3.80	20.9	±	1.31	17.8	26.2	6.28	17.1		±	1.23	15.9	21.1
**uNDFom (% aNDF)**	52.1	±	0.59	49.9	53.7	1.13	54.1	±	0.76	50.9	55.7	1.41	56.1		±	0.59	54.1	56.6
**uNDFom (% DM)**	16.9	±	0.57	15.0	18.5	3.36	19.1	±	0.80	15.8	20.9	4.20	21.4		±	0.75	18.9	22.1

^a^Alfalfa hay: 91.8% DM, 51.3% aNDF, 6.37% ADL, 13.4% CP.

^b^Concentrate: 91.3% DM, 19.3 aNDF, 2.33% ADL, 41.2% Starch, 16.8% CP.

For the determination of AIA, 5 g of sample were burned on a Bunsen burner and subsequently boiled in a hotplate containing 2N HCl for 15 min [11,22]. The residue was then filtered (Whatman No 41), transferred in a porcelain crucible, and ashed by ignition at 550°C according to the European Commission Regulation No.152/2009 [23].

The ttaD of DM (ttaDMD), CP (ttaCPD), and NDF soluble CP (ttaNDFSolCPD), and the ttD of NDF (ttNDFD), ADF (ttADFD), hemicellulose (ttHemicelDe), cellulose (ttCelD), NDICP (ttNDICPD), ADICP (ttADICPD) ash (ttAshD) and pdNDF (ttpdNDFD) were calculated using the individual dietary markers (uNDF, uNDFom and AIA) concentration and the proportion of the markers of the related faecal samples as described by [2,11].

### NIRS spectra collection and calibration development

NIRS spectra were obtained from 30 g aliquots of pre-dried and ground faecal samples. Spectra from different species and animals consuming diets with varying forage to concentrate ratios were combined in a single calibration curve to improve data variability, thereby improving the robustness of the calibrations [24]. Each sample was placed in a 40 mm diameter glass cup and scanned at room temperature using the NIRS DS2500 instrument (FOSS, Electric A/S, Hillerød, Denmark) covering a wavelength range of 400–2500 nm, with a spectral resolution of 0.5 nm. The resulting spectrum was an average of 32 sub-spectra recorded at different locations on the sample surface.

Regression models were developed using modified partial least squares (mPLS) regression analysis with WinISI 4 software (Infrasoft International, Port Matilda, PA) to correlate spectral data with reference values. Spectral outliers were identified based on Mahalanobis distance, excluding samples from the calibration dataset if their Global H value exceeded 3.0 from the mean spectrum. To enhance model accuracy, different combinations of spectral pre-treatments were evaluated. Various scatter correction methods were employed to reduce noise, including detrending (D), standard normal variate (SNV), SNV combined with detrending (SNV + D), and multiplicative scatter correction (MSC) [25]. Additionally, spectral derivatization was applied using different parameters combinations: (0,0,1,1; 1,4,4,1; 1,8,8,1; 2,5,5,1; 2,10,10,1). The first digit represents the order of the derivative, the second digit indicates the gap over which the derivative was computed, the third digit specifies the number of data points used for smoothing the derivative spectra, and the fourth digit denotes the number of data points used for the second smoothing [26].

Prediction equations were validated using both a 5-fold cross-validation and an external validation procedure. For cross-validation, the dataset was divided into five random, representative subsets: four subsets were used to train the model, and the remaining subset was used for validation. This process was repeated iteratively until each subset had been used once for validation. Additionally, a calibration set consisting of 75% of the samples was randomly selected from the full dataset to develop the final prediction equation, which was subsequently validated using the remaining 25% of samples. In both cross- and external validation, steps were taken to ensure that the calibration and validation subsets for each trait had comparable means and standard deviations. The optimal number of latent factors (LF) was determined to minimize the root mean square error of cross-validation, to minimize the risk of overfitting. Furthermore, three iterations of chemical outlier detection were conducted using the T-statistic (> 3), resulting in the removal of samples with predicted values deviating more than 3 standard errors from the reference value.

Identification of the optimal calibration models was based on several metrics: the standard error of cross-validation (SE_CrV_) and external validation (SE_ExV_), the coefficient of determination for cross-validation (R²_CrV_) and external validation (R²_ExV_), and the residual predictive deviation (RPD) of external validation. The RPD was calculated as the ratio of the standard deviation to SE_ExV_, facilitating comparison of model performance across different units of measurement. A value of R^2^_ExV_ < 0.66 was deemed indicative of inadequate prediction accuracy. An R value of 0.66 to 0.81 suggests that the prediction may provide an approximate estimate of the reference value. Values of R^2^_ExV_ between 0.82 and 0.90 are considered to indicate a good prediction, while R^2^_ExV _> 0.90 indicates excellent estimation accuracy [27]. The classification criteria for the RPD as described by [28] are as follows: an RPD value of less than 1.0 denotes a very poor model with unreliable predictions, making it unsuitable for practical use. RPD values ranging from 1.0 to 1.4 suggest poor model performance, where only broad distinctions between high and low values can be made. Values between 1.4 and 1.8 indicate a fair or moderately good model. An RPD value between 1.8 and 2.0 suggests a good model where quantitative predictions are feasible. RPD values between 2.0 and 2.5 reflect a very good model capable of reliable quantitative predictions, while values above 2.5 are indicative of an excellent model with highly reliable predictions.

### Statistical analysis for digestibility comparison

Statistical analyses were performed using GraphPad Prism version 10.0.0 for Windows (GraphPad Software, Boston, Massachusetts, USA). Normality was assessed graphically and by using the Shapiro–Wilk test. Since digestibility of nutrient were not normally distributed, a Dunn’s multiple comparison test was used to compare digestibility values for each nutrient determined by total collection (TC) or estimated with markers technique (TC vs uNDF vs uNDFom vs AIA). Linear regressions models were applied to evaluate the markers performance plotting the markers excretion (y; g/day) over their intake (x; g/day). Furthermore, a Sperman’s correlation was utilized to evaluate the relationship between the intake and the excreted undegradable materials, for uNDF, uNDFom and AIA.

## Results

### Chemical composition of the diets

The calculated chemical composition of the diet fed to dairy sheep and goats offered alfalfa hay and concentrate separately at different F:C ratios is in Table 1. As expected, increasing the F:C ratio, the calculated level of aNDFom, ADFom and uNDFom increased by about 6, 5 and 4% respectively, while the level of starch decreased by more than 7%. The variation in CP, soluble CP, and ether extract was lower than that observed for the NDF and starch contents.

The descriptive statistics of the average chemical composition of the faeces collected from dairy sheep and goats can be found in our previous work [13].

### Total-tract apparent and true digestibility estimated by uNDF, uNDFom and AIA

Descriptive statistics of the ttaD and ttD estimated through uNDF, uNDFom and AIA are in Tables 2,3,4 respectively; a comparison between the average values is in Table 5.

**Table 2:** Descriptive statistics of average total-tract apparent (tta) and total-tract (tt) nutrients digestibility (D) estimated using faecal uNDF as a marker in faeces of small ruminants.

Trait^a^	n	Mean	SD^b^	Min	Max	CV^c^
**ttaDMD**	180	63.8	5.12	39.1	74.2	8.03
**ttNDFD**	180	37.2	6.40	13.7	50.3	17.1
**ttNDICPD**	179	39.2	10.1	6.35	63.0	25.8
**ttADFD**	180	34.6	6.81	11.0	46.9	19.6
**ttADICPD**	180	80.1	5.09	52.5	89.5	6.35
**ttHemicelD**	179	42.5	7.40	18.8	80.0	17.2
**ttCelD**	180	62.5	12.8	40.7	95.5	20.5
**ttaCPD**	180	60.3	6.39	25.4	72.1	10.6
**ttaSolCPD**	180	64.7	6.06	32.5	77.3	9.36
**ttAshD**	143	20.2	12.8	0.40	85.4	6.32
**ttpdNDFD**	180	74.5	12.3	27.8	98.5	16.6

^a^ttaDMD = total-tract apparent dry matter digestibility; ttAshD = total-tract ash digestibility; ttaCPD = total-tract apparent crude protein digestibility; ttaSolCPD = total-tract apparent soluble crude protein digestibility; ttNDFD = total-tract amylase treated neutral detergent fibre; ttNDICPD = total-tract neutral detergent insoluble crude protein digestibility; ttADFD = total-tract acid detergent fibre digestibility; ttADICPD = total-tract acid detergent insoluble crude protein digestibility; ttHemicelD = total-tract hemicellulose digestibility; ttCelD = total-tract cellulose digestibility; ttpdNDFD = total-tract potentially degradable amylase treated neutral detergent fibre.

^b^SD = standard deviation.

^c^CV = coefficient of variation.

**Table 3:** Descriptive statistics of average total-tract apparent (tta) and total-tract (tt) nutrients digestibility (D) estimated using faecal uNDFom as a marker in faeces of small ruminants.

Trait^a^	n	Mean	SD^b^	Min	Max	CV^c^
**ttaDMD**	180	61.8	4.40	51.5	71.6	7.10
**ttNDFD**	180	33.7	5.00	11.0	45.8	14.9
**ttNDICPD**	180	35.6	10.5	8.50	59.4	29.6
**ttADFD**	180	31.0	5.40	10.8	42.3	17.2
**ttADICPD**	180	79.0	5.00	62.6	88.6	6.30
**ttHemicelD**	179	39.6	6.98	22.1	78.2	17.5
**ttCelD**	180	60.4	13.2	40.1	95.3	21.9
**ttaCPD**	180	58.1	5.84	40.1	70.8	10.0
**ttaSolCPD**	180	62.8	5.56	43.8	75.1	8.80
**ttAshD**	125	17.5	11.8	0.50	84.8	67.8
**ttpdNDFD**	179	73.6	10.8	25.1	95.7	14.7

^a^ttaDMD = total-tract apparent dry matter digestibility; ttAshD = total-tract ash digestibility; ttaCPD = total-tract apparent crude protein digestibility; ttaSolCPD = total-tract apparent soluble crude protein digestibility; ttNDFD = total-tract amylase treated neutral detergent fibre; ttNDICPD = total-tract neutral detergent insoluble crude protein digestibility; ttADFD = total-tract acid detergent fibre digestibility; ttADICPD = total-tract acid detergent insoluble crude protein digestibility; ttHemicelD = total-tract hemicellulose digestibility; ttCelD = total-tract cellulose digestibility; ttpdNDFD = total-tract potentially degradable amylase treated neutral detergent fibre.

^b^SD = standard deviation.

^c^CV = coefficient of variation

**Table 4:** Descriptive statistics of average total-tract apparent (tta) and total-tract (tt) nutrients digestibility (D) estimated using faecal AIA as a marker in faeces of small ruminants.

Trait^a^	n	Mean	SD^b^	Min	Max	CV^c^
**ttaDMD**	179	64.1	11.4	22.0	91.5	17.8
**ttNDFD**	175	38.4	19.1	2.85	85.3	49.8
**ttNDICPD**	176	40.7	18.3	3.54	84.1	44.9
**ttADFD**	172	36.4	20.0	0.31	84.4	54.9
**ttADICPD**	177	80.4	7.20	47.7	95.9	9.00
**ttHemicelD**	179	43.6	17.8	4.49	87.0	40.8
**ttCelD**	179	62.2	18.6	13.9	95.9	29.8
**ttaCPD**	179	60.7	12.5	15.1	90.2	20.6
**ttaSolCPD**	179	65.1	11.3	27.9	91.4	17.3
**ttAshD**	129	26.9	19.0	0.06	82.3	70.7
**ttpdNDFD**	179	75.1	13.7	12.7	97.9	18.3

^a^ttaDMD = total-tract apparent dry matter digestibility; ttAshD = total-tract ash digestibility; ttaCPD = total-tract apparent crude protein digestibility; ttaSolCPD = total-tract apparent soluble crude protein digestibility; ttNDFD = total-tract amylase treated neutral detergent fibre; ttNDICPD = total-tract neutral detergent insoluble crude protein digestibility; ttADFD = total-tract acid detergent fibre digestibility; ttADICPD = total-tract acid detergent insoluble crude protein digestibility; ttHemicelD = total-tract hemicellulose digestibility; ttCelD = total-tract cellulose digestibility; ttpdNDFD = total-tract potentially degradable amylase treated neutral detergent fibre.

^b^SD = standard deviation.

^c^CV = coefficient of variation.

**Table 5:** Comparison of the descriptive statistics of average total-tract apparent (tta) and total-tract (tt) nutrients digestibility (D) determined by total collection (TC) and estimated using faecal uNDF, uNDFom and AIA as a marker in faeces of small ruminants.

Trait^1^	TC		uNDF		uNDFom		AIA		SEM	p-Value
**ttaDMD**	71.6	a^2^	63.7	b	61.8	c	64.1	bc	0.488	<0.0001
**ttNDFD**	50.4	a	37.2	b	33.8	c	38.4	b	0.763	<0.0001
**ttNDICP**	52.2	a	39.3	b	35.6	c	40.7	b	0.912	<0.0001
**ttADFD**	48.3	a	34.7	b	31.1	c	36.4	b	0.804	<0.0001
**ttADICP**	84.5	a	80.2	bc	79.1	c	80.4	b	0.392	<0.0001
**ttHemicelD**	54.1	a	42.9	b	39.7	c	43.6	b	0.786	<0.0001
**ttCelD**	70.2	a	62.5	b	60.4	b	62.2	b	1.051	<0.0001
**ttaCPD**	68.8	a	60.3	c	58.1	c	60.7	b	0.578	<0.0001
**ttaSolCPD**	72.3	a	64.7	b	62.8	c	65.1	b	0.539	<0.0001
**ttAshD**	33.1	a	20.3	c	17.5	c	26.9	b	1.221	<0.0001
**ttpdNDFD**	80.3	a	74.6	b	73.6	b	75.2	b	0.864	<0.0001

^1^ttaDMD = total-tract apparent dry matter digestibility; ttAshD = total-tract ash digestibility; ttaCPD = total-tract apparent crude protein digestibility; ttaSolCPD = total-tract apparent soluble crude protein digestibility; ttNDFD = total-tract amylase treated neutral detergent fibre; ttNDICPD = total-tract neutral detergent insoluble crude protein digestibility; ttADFD = total-tract acid detergent fibre digestibility; ttADICPD = total-tract acid detergent insoluble crude protein digestibility; ttHemicelD = total-tract hemicellulose digestibility; ttCelD = total-tract cellulose digestibility; ttpdNDFD = total-tract potentially degradable amylase treated neutral detergent fibre.

^2^(a-d) Different lowercase letter within a row are statistically different (p < 0.001).

Concerning the digestibility estimated through the uNDF, the average ttaDMD, ttADICPD, ttCelD, ttaSolCPD, and ttpdNDFD were above 60.3%, whereas ttHemicelD was on average 42.5%. The means of all the other parameters were lower than 39.2%, and the variability ranged between 6.32% (ttADICPD) and 25.8% (ttNDICPD).

Regarding the estimations made using the uNDFom marker, the average values for ttaDMD, ttADICPD, ttCelD, ttaSolCPD, and ttpdNDFD exceeded 60.4%, whereas ttaCPD averaged 58.1%. All the other parameters had means below 39.6%, with variability ranging from 6.30% (ttADICPD) to 67.8% (ttaAshD).

Concerning the digestibility estimated using AIA, the average ttaDMD, ttADICPD, ttCelD, ttaCPD, ttaSolCPD, and ttpdNDFD were above 60.7%. The means of all the other parameters were lower than 40.7%, and the variability ranged between 9.0% (ttADICPD) and 70.7% (ttAshD).

Values of nutrient digestibility of the sheep and goats recruited in the present study obtained with the TC technique are reported in Table 5 where the same values where also compared with those estimated using faecal uNDF, uNDFom, and AIA as internal markers. In comparison to the TC data, the use of markers significantly underestimated the digestibility of all the nutrients. The ttaD and ttD results obtained using the uNDF and the AIA markers are generally similar, with the exception of ttaCPD and ttAshD, which were estimated as significantly lower using uNDF (60.3 vs 60.7% and 20.3 vs 26.9% respectively; p < 0.0001), being similar to those obtained using uNDFom. Overall, the latter marker gave the lowest digestibility estimates for all the nutrients, with ttaDMD similar the one obtained from AIA, and ttCelD and ttpdNDFD similar to those estimated by both uNDF and AIA.

### NIRS evaluation of nutrients digestibility

The performance statistics of the prediction models for the ttaD and ttD estimated using uNDF, uNDFom or AIA from faecal spectra are in Table 6, 7 and 8, respectively. For the development of the most accurate calibration, the detrend, alone or combined with SNV, was the main scatter correction used, while the first derivative was the mathematical treatment more frequently employed.

**Table 6:** Fitting statistics of modified partial least square regression models using external validation for total-tract apparent (tta) and total-tract (tt) nutrients digestibility estimated (D) using faecal uNDF as a marker in faeces of small ruminants.

Trait^a^	n_CrV_	LF^b^	SE_CrV_^c^	R^2^_CrV_^d^	n_ExV_	SE_ExVe_^e^	R^2^_ExV_^f^	BIAS	SLOPE	RPD_ExV_^g^
**ttaDMD**	122	8	2.95	0.53	45	3.53	0.44	1.08	0.98	1.34
**ttNDFD**	114	9	3.88	0.37	45	4.88	0.20	−1.01	0.63	1.07
**ttNDICPD**	130	6	6.02	0.46	45	8.00	0.20	3.30	0.56	1.04
**ttADFD**	116	3	3.94	0.29	44	5.22	0.28	1.72	0.82	1.17
**ttADICPD**	124	9	2.67	0.60	44	2.92	0.63	−0.45	0.80	1.56
**ttHemicelD**	108	4	4.67	0.07	45	7.13	0.08	−0.26	0.49	1.00
**ttCelD**	110	1	10.7	0.06	44	10.7	0.07	2.24	0.70	1.03
**ttaCPD**	124	6	3.64	0.43	39	3.48	0.47	0.21	0.91	1.37
**ttaSolCPD**	118	8	3.69	0.45	44	3.90	0.29	3.70	0.55	1.06
**ttAshD**	86	5	8.30	0.52	40	7.11	0.59	0.33	0.77	1.45
**ttpdNDFD**	117	4	6.98	0.26	40	9.07	0.22	0.38	0.59	1.06

^a^ttaDMD = total-tract apparent dry matter digestibility; ttAshD = total-tract ash digestibility; ttaCPD = total-tract apparent crude protein digestibility; ttaSolCPD = total-tract apparent soluble crude protein digestibility; ttNDFD = total-tract amylase treated neutral detergent fibre; ttNDICPD = total-tract neutral detergent insoluble crude protein digestibility; ttADFD = total-tract acid detergent fibre digestibility; ttADICPD = total-tract acid detergent insoluble crude protein digestibility; ttHemicelD = total-tract hemicellulose digestibility; ttCelD = total-tract cellulose digestibility; ttpdNDFD = total-tract potentially degradable amylase treated neutral detergent fibre.

^b^LF = latent factors.

^c^SE_CrV_ = standard error of cross-validation.

^d^R^2^_CrV_ = coefficient of determination of cross-validation.

^e^SE_ExV_ = standard error of external validation.

^f^R^2^_ExV_= coefficient of determination of external validation.

^g^RPD_ExV_ = residual predictive deviation of external validation

**Table 7:** Fitting statistics of modified partial least square regression models using external validation for total-tract apparent (tta) and total-tract (tt) nutrients digestibility estimated (D) using faecal uNDFom as a marker in faeces of small ruminants.

Trait^a^	n_CrV_	LF^b^	SE_CrV_^c^	R^2^_CrV_^d^	n_ExV_	SE_ExVe_^e^	R^2^_ExV_^f^	BIAS	SLOPE	RPD_ExV_^g^
**ttaDMD**	123	5	2.77	0.54	45	3.33	0.55	1.25	1.03	1.42
**ttNDFD**	118	3	4.59	0.24	39	5.35	0.10	1.14	0.42	0.96
**ttNDICPD**	132	8	4.20	0.49	39	9.17	0.17	−1.12	0.52	1.02
**ttADFD**	110	4	4.35	0.31	44	5.52	0.26	2.58	0.77	1.05
**ttADICPD**	125	9	2.03	0.67	45	2.69	0.62	−0.33	0.62	1.61
**ttHemicelD**	109	1	5.91	0.06	45	7.79	0.08	0.33	−0.08	0.87
**ttCelD**	106	1	10.2	0.10	44	15.7	0.02	4.16	−0.23	0.45
**ttaCPD**	140	5	4.66	0.38	39	3.83	0.43	1.52	0.81	1.30
**ttaSolCPD**	138	7	3.50	0.44	34	4.02	0.22	1.13	0.56	1.02
**ttAshD**	96	7	7.77	0.52	30	7.54	0.46	0.10	1.05	1.36
**ttpdNDFD**	113	2	9.41	0.09	39	9.47	0.24	3.92	1.23	1.14

^a^ttaDMD = total-tract apparent dry matter digestibility; ttAshD = total-tract ash digestibility; ttaCPD = total-tract apparent crude protein digestibility; ttaSolCPD = total-tract apparent soluble crude protein digestibility; ttNDFD = total-tract amylase treated neutral detergent fibre; ttNDICPD = total-tract neutral detergent insoluble crude protein digestibility; ttADFD = total-tract acid detergent fibre digestibility; ttADICPD = total-tract acid detergent insoluble crude protein digestibility; ttHemicelD = total-tract hemicellulose digestibility; ttCelD = total-tract cellulose digestibility; ttpdNDFD = total-tract potentially degradable amylase treated neutral detergent fibre.

^b^LF = latent factors.

^c^SE_CrV_ = standard error of cross-validation.

^d^R^2^_CrV_ = coefficient of determination of cross-validation.

^e^SE_ExV_ = standard error of external validation.

^f^R^2^_ExV_= coefficient of determination of external validation.

^g^RPD_ExV_ = residual predictive deviation of external validation

**Table 8:** Fitting statistics of modified partial least square regression models using external validation for total-tract apparent (tta) and total-tract (tt) nutrients digestibility estimated (D) using faecal AIA as a marker in faeces of small ruminants.

Trait^a^	n_CrV_	LF^b^	SE_CrV_^c^	R^2^_CrV_^d^	n_ExV_	SE_ExVe_^e^	R^2^_ExV_^f^	BIAS	SLOPE	RPD_ExV_^g^
**ttaDMD**	111	1	11.2	0.00	44	5.57	0.07	−0.15	0.36	1.05
**ttNDFD**	115	2	18.5	0.06	43	19.2	0.02	−2.24	0.33	0.97
**ttNDICPD**	110	2	18.3	0.01	44	18.3	0.03	1.12	0.64	1.00
**ttADFD**	104	2	19.4	0.02	43	20.4	0.01	2.23	0.40	0.97
**ttADICPD**	117	1	7.01	0.12	44	6.04	0.20	0.33	1.38	1.10
**ttHemicelD**	115	1	17.5	0.06	44	18.8	0.02	−0.95	0.28	0.96
**ttCelD**	115	2	17.5	0.09	44	17.3	0.10	−4.34	0.68	1.04
**ttaCPD**	112	2	12.2	0.00	44	12.7	0.00	1.25	0.12	0.92
**ttaSolCPD**	115	1	11.0	0.00	44	11.1	0.00	−1.14	−0.03	0.98
**ttAshD**	82	1	18.8	0.03	32	20.4	0.00	3.19	0.08	0.94
**ttpdNDFD**	122	5	11.7	0.22	44	12.2	0.18	−0.31	0.56	1.09

^a^ttaDMD = total-tract apparent dry matter digestibility; ttAshD = total-tract ash digestibility; ttaCPD = total-tract apparent crude protein digestibility; ttaSolCPD = total-tract apparent soluble crude protein digestibility; ttNDFD = total-tract amylase treated neutral detergent fibre; ttNDICPD = total-tract neutral detergent insoluble crude protein digestibility; ttADFD = total-tract acid detergent fibre digestibility; ttADICPD = total-tract acid detergent insoluble crude protein digestibility; ttHemicelD = total-tract hemicellulose digestibility; ttCelD = total-tract cellulose digestibility; ttpdNDFD = total-tract potentially degradable amylase treated neutral detergent fibre.

^b^LF = latent factors.

^c^SE_CrV_ = standard error of cross-validation.

^d^R^2^_CrV_ = coefficient of determination of cross-validation.

^e^SE_ExV_ = standard error of external validation.

^f^R^2^_ExV_= coefficient of determination of external validation.

^g^RPD_ExV_ = residual predictive deviation of external validation.

Concerning uNDF, latent factors ranged from 1 (ttCelD) to 9 (ttNDFD). The greater accuracy was reached for ttADICP (R^2^_ExV_ = 0.63, RPD = 1.56), whereas the worst results were obtained for ttHemicelluloseD, and ttCelluloseD (R^2^_ExV_ < 0.07, RPD < 1.00).

Considering uNDFom, latent factors ranged from 1 (ttHemicelD and ttCelluloseD) to 9 (ttADICPD). The greater accuracies were reached for ttADICP (R^2^_ExV_ = 0.62, RPD = 1.61) and for ttaDMD (R^2^_ExV_ = 0.55, RPD = 1.42), whereas the worst result was obtained for ttCelD (R^2^_ExV_ = 0.02, RPD = 0.45).

Lastly, AIA’s latent factors ranged from 1 (ttaDMD, ttADICP, ttHemicelD, ttaSolCPD, ttAsh) to 5 (ttpdNDFD). The greater accuracy was reached for ttADICP (R^2^_ExV_ = 0.20, RPD = 1.10) and for ttpdNDFD (R^2^_ExV_ = 0.18, RPD = 1.09), whereas all other results obtained worst predictive performance (R^2^_ExV_ < 0.10, RPD < 1.05).

The regression coefficients reported in Table 9 indicate the strength and nature of the relationship between the intake and the excretion of the studied markers. Results indicates that uNDF had a low relationship between intake and excretion (R^2^ = 0.290, slope 0.609), while uNDFom had a higher R^2^ and slope (R^2^ = 0.372, slope 0.630), which may indicate that uNDFom provides a more consistent estimation of excretion across different intake levels. In contrast, AIA demonstrated a very weak and unreliable relationship (R^2^ = 0.009, slope 0.262), making it less dependable for predicting nutrient excretion. Spearman correlation coefficients between intake and excretion remain consistent with the regression trend, with uNDF having a lower coefficient compared to uNDFom (0.548 vs 0.578), and AIA following on a lower level (0.189).

**Table 9:** Regression and correlation coefficients of the recovery, as excretion (y; g/day) plotted against its intake (x; g/day), for uNDF, uNDFom and AIA.

	Regression			Correlation
Marker^1^	R^2^	Intercept	Slope; p	r; p
**uNDF (kg/kg)**	0.290	59.44	0.609; p < 0.001	0.548; < 0.001
**uNDFom (kg/kg)**	0.372	41.41	0.630; p < 0.001	0.578; < 0.001
**AIA (kg/kg)**	0.009	2.638	0.262; p = 0.195	0.189; 0.011

## Discussion

### Total-tract apparent and true digestibility

The use of internal faecal markers to estimate ttaD and ttD of dietary nutrients offers a convenient alternative to the TC technique in digestibility trials [29]. Various markers can be used with different levels of effectiveness, raising the question of which marker performs the closest to digestibility calculated by TC [30].

From our results, it appears that measurements of nutrient digestibility obtained by TC are consistently higher compared to the estimates performed using faecal uNDF, uNDFom, and AIA. This discrepancy could be attributed to the recovery rates of the markers, which inherently varied based on their chemical nature [31]. In our study, the recovery rates were 79.5 ± 16.5, 74.9 ± 14.9 and 72.5 ± 27.9% for uNDF, uNDFom, and AIA respectively. The recovery of uNDF statistically differed from the recovery of AIA, while the recovery of uNDFom was similar to both of them. Both uNDF and uNDFom performed better than AIA in terms of slope and correlation between markers’ input and output from the animals, possibly as consequence of their higher concentration in diets and faeces, and consequently their more accurate measurement in these substrates [32].

Consistent with our digestibility results, a study reported ttaDMD estimated by indigestible NDF marker (iNDF, comparable to uNDF), resulted in lower values of digestibility compared to those by TC (61.6 vs 75.4%, respectively) in lambs [30]. Similarly, using uDM and uNDF as markers, correlation coefficients of 0.793 and 0.806 respectively were found between TC and the estimated ttaDMD. Specifically, the ttaDMD was overestimated (+2.5%) and underestimated (−2.2%) using uDM and uNDF markers respectively compared to TC [4].

In another sheep and goat study, various internal markers, like indigestible DM (iDM), iNDF, and iADF, were evaluated for their effectiveness in estimating digestibility in comparison to TC [32]. Although no bias in digestibility was detected in the long-term for any of the markers employed, and the markers performed similarly in estimating ttaDMD with the TC technique, iNDF resulted to be the most suitable and reliable marker when the data were analysed independently of animal species [33].

In a very recent work [34], the ttaDMD of sheep and goat diets were estimated using iNDF, iADF or iDM at 244 h or 264 hours, and compared to TC. Overall, it was concluded that iNDF was the marker that more accurately estimated ttaDMD in both species, while the estimation performed through iDM measured at 264 h statistically differed from the TC data in all the diets employed. Additionally, the ttaDMD from total faecal collection, differed by 3.3% (75.9 vs 71.6%), while the ttaDMD estimated with uNDF, differed by 12.9% (63.7 vs 76.7%) when our results were compared with those of the aforementioned study. These differences could however be related to the marker recovery and the methodological approach used for the digestibility testing.

It is worth noting, as suggested by some authors [35], that when long-term incubation tests have to be performed to measure uNDF and uNDFom on the same feeds in both sheep and cattle, a longer incubation period may be required for sheep. This suggests that markers such as uNDF and uNDFom, developed based on cattle degradability models, including the incubation timepoint and the rumen inoculum employed, may not be entirely reliable for small ruminants due to differences in their gastrointestinal physiology.

### NIRS prediction models of total-tract apparent and true nutritional digestibility

Considering NIRS predictive models for ttaDMD by TC in small ruminants, a recent study reported good calibrations equations, although they were insufficient for highly accurate predictions (R^2^_ExV_ = 0.60; RPD_ExV_ = 1.46) [36]. This supports the idea that ttaDMD calculated by TC can be reasonably predicted using NIRS. In contrast, a recent investigation from [13], reported unsatisfactory calibration results for ttaDMD (R^2^_ExV_ = 0.34; RPD_ExV_ = 1.22), whereas better calibrations models were achieved for ttADICPD (R^2^_ExV_ = 0.57; RPD_ExV_ = 1.38). To the best of our knowledge, no studies have modelled digestibility estimation using faecal internal markers, such as uNDF, uNDFom, and AIA with NIRS predictions in small ruminants.

The performance of the regression models for ttaD and ttD, using faecal uNDF as a marker of dried faeces in small ruminants, showed mixed results. For ttaDMD, the R^2^_ExV_ value was 0.44, indicating an insufficient accuracy for prediction, with an RPD_ExV_ of 1.34, classifying the model as poor. Similarly, ttNDICPD model showed a low predictive ability, with an R^2^_ExV_ of 0.20 and an RPD_ExV_ of 1.04, further confirming poor model performance. The best results were observed for ttaADICPD, with a value of R^2^_ExV_ of 0.63 and an RPD_ExV_ of 1.56, and for ttAshD, with an R^2^_ExV_ of 0.59 and an RPD_ExV_ of 1.45. These values only suggest moderately good performance. Overall, the models using faecal uNDF mostly provided insufficient accuracy and poor reliability, making them suitable only for screening purposes.

When using faecal uNDFom as a marker, the models again showed mostly insufficient predictive performance. The ttaDMD had an R^2^_ExV_^f^ of 0.55 and an RPD_ExV_^g^ of 1.42, indicating a model which could be used only for rough screening. For ttCelD, the R^2^_ExV_ was 0.02 with an RPD_ExV_ of 0.45, reflecting the poorest prediction. The best performance for uNDFom was observed in the ttADICPD model, with an R^2^_ExV_ of 0.62 and an RPD_ExV_ of 1.61, suggesting that this model may be marginally suitable for rough screening and classified as moderately good. In general, predictive accuracy using uNDFom remained low, with most models being classified as poor.

The models using faecal AIA as a marker exhibited the weakest performance among the three methods evaluated in this study. All digestibility traits assessed using AIA as a marker showed poor predictive performance, with a value of R^2^_ExV_ < 0.10 and RPD_ExV_ < 1.05, which are below the threshold for even rough screening. An exception was observed for ttADICP and for ttpdNDFD, that exhibited a value of R^2^_ExV_ 0.20 and 0.18, respectively.

Thus, the models using AIA cannot be recommended for the practical use due to their insufficient accuracy and poor predictive ability. Therefore, while none of the markers provided highly accurate predictions, faecal uNDF and uNDFom were the most reliable for screening digestibility traits in small ruminants. The predictive performance, particularly for digestibility traits with medium values of R²_ExV_ and RPD approaching acceptable thresholds, could potentially be enhanced by increasing the sample size and replicating sampling days. This approach would improve variability and, consequently, model accuracy.

Overall, the results of the present study show that, despite convenient from a practical perspective, the use of markers provides only approximate estimates of the nutrients digestibility in sheep and goats fed forage and concentrate separately. This evidence, which is consistent with an incomplete recovery of the markers and, in this specific study, to criticisms related to their determination, represents *per se* a limitation in their possible use for NIRS calibration. Additionally, faecal NIRS spectra seems unable to provide enough information to lead to an accurate prediction of the diet’s total-tract digestibility because the complete nutritive profile of the diet is not taken into consideration since the spectra relates to the undigested residues of faeces [13]. Moreover, the low variability of the diets and consequently of the excreted faeces, along with a moderate number of samples considered in the present study, could have contributed in reducing the NIRS calibration performances.

## Conclusions

All the markers considered, namely uNDF, uNDFom and AIA, tended to underestimate total tract digestibility compared to the TC technique. The nutrients digestibility estimated by uNDFom were lower than those estimated by uNDF and AIA, and the latter marker was characterized by the lowest recovery in comparison to the others. The NIRS technology seemed to perform slightly better when the faecal uNDF and uNDFom, instead of AIA, were employed to estimate digestibility, and can be used, depending on the parameter considered, for screening digestibility traits in small ruminants.
